# 

**Published:** 1900-01

**Authors:** 


### CONTENTS.

ORIGINAL COMMUNICATIONS—

THE PHYSIOLOGICAL THERAPEUTICS OF REASONING MADNESS. By C. A. F. Lindorme, Ph.D., M.D., Chicago, Ill. .... 721
SOME NOTES ON THE PHILIPPINES. By Frank S. Bourns, M.D., Atlanta, Ga. .... 729
DIET IN LITHEMIA. By A. B. Conklin, M.D. .... 734
A PAINLESS APPLICATION OF ARSENICAL PASTE. By William Perrin Nicolson, M.D., Atlanta, Ga. .... 738

SOCIETY REPORTS—

SOUTHERN SURGICAL AND GYNECOLOGICAL ASSOCIATION .... 740

EDITORIAL NOTES AND COMMENTS—

THE RISE AND FALL OF OSTEOPATHY IN GEORGIA .... 758
THE SOUTHERN SURGICAL AND GYNECOLOGICAL ASSOCIATION .... 761

NEWS AND NOTES .... 766
NEW YORK NOTES .... 771

CONTENTS CONTINUED .... x

